# Phenotypic and genotypic screening of multidrug resistant *Klebsiella pneumoniae* isolated from ready to eat street food in Tanta, Egypt

**DOI:** 10.1186/s12866-025-03769-z

**Published:** 2025-02-05

**Authors:** Hadeer M. Bedair, Mohamed Emara, Shima Mahmoud Ali, Tamer M. Samir, Mahmoud A.F. Khalil

**Affiliations:** 1 Department of Microbiology and Immunology, Faculty of Pharmacy, Helwan University − Ain Helwan, Helwan, 11795 Egypt; 2 Department of Microbiology and Immunology, College of Pharmaceutical sciences and drug manufacturing, Misr University for Science and technology, Cairo, 12566 Egypt; 3 Department of Microbiology and Immunology, Faculty of Pharmacy, Fayoum University, Fayoum, 63514 Egypt

**Keywords:** *Klebsiella pneumoniae*, Multiplex PCR, MDR, Foodborne, Hodge, Biofilm

## Abstract

**Background:**

Ready-To-Eat-Street-Foods (RTESF) have food safety concerns, since they are prepared with less-structured food safety guidelines in small and roadside outlets. *Klebsiella pneumoniae* has become a dangerous foodborne-pathogen worldwide due to its virulence and resistance profile.

**Objective:**

This study aimed at evaluating the potential burden of antibiotic-resistant *Klebsiella pneumoniae* contaminating RTESF and assessing the microbiological quality of RTESF in Egypt.

**Methods:**

A total of 242 RTESF (green salad) samples was collected, different media were used for isolation of different bacterial species. *Klebsiella pneumoniae* isolates were identified biochemically and by Gram and capsular staining then isolates were assessed for antimicrobial resistance phenotypically. The ability of biofilm formation was assessed using crystal violet and molecular characterization of ESBLs and virulence genes was done using PCR.

**Results:**

A total of 77/242(31.8%) of the recovered isolates was identified as *Klebsiella pneumoniae *and the resistance percentages were as follow: cefuroxime and cephradine (100%, 77/77), amoxicillin-clavulanic acid (98.7%, 76/77), while (27.3%, 21/77) of the isolates were MDR. Biofilm assay revealed that (31/77, 41/77 and 5/77) isolates were strong, moderate, and weak biofilm-producers, respectively. Among ESBLs-encoding-genes, *bla*_SHV_ was the most prevalent (71.4%) while *bla*_TEM _and *bla*_CTX−M−2_were equally-present (55.8%).The most prevalent virulence genes were *mrkD* (92.2%) followed by *K2 (*63.3%).

**Conclusion:**

The contaminated RTESF could be a reservoir for *Klebsiella pneumoniae*, therefore much care must be taken during preparation and consumption to avoid dissemination of MDR *Klebsiella pneumoniae* leading to subsequent treatment challenges. Our finding indicating that RTESF, if not prepared under hygienic conditions, could be a source of serious *Klebsiella pneumoniae* infection.

**Supplementary Information:**

The online version contains supplementary material available at 10.1186/s12866-025-03769-z.

## Background

Ready-To-Eat-Street-Foods (RTESF) are defined as foods for immediate consumption or use with no need for further processing or preparation and are sold as common street foods in small roadside outlets [1]. There is a higher dependency on this type of food due to convenience and acceptance by consumers additionally, RTESF save time and considered inexpensive [1, 2]. Unfortunately, the consumption of street food increases the potential risk of foodborne illnesses such as diarrhea or traveler’s diarrhea [1]. FBDs are considered a public health concern in many countries as contaminated food was reported to be responsible for up to 600 million FBDs and an estimated global burden of up to 33 million disability-adjusted life years [3]. Moreover, more than 91 million African people are affected by foodborne diseases according to the report by the World Health Organization (WHO) [4]. In Africa, the prevalence of food poisoning is underestimated, since people with gastrointestinal symptoms rarely go to health facilities [4].

Salads are considered minimally processed foods because they only undergo washing, peeling, chopping, drying, and packaging—no heat treatment [5]. In addition to that, processes like cutting and peeling might disrupt their exterior natural barrier and release plant juices that encourage microbial growth, so fruits and vegetables are more vulnerable to microbial contamination and proliferation [6].It was reported that vegetables have also been implicated as carriers of foodborne pathogens, such as *Salmonella* species, *Campylobacter* species, *Listeria monocytogenes*, and enterohaemorrhagic strains of *Escherichia coli* [7]. These pathogens can affect vegetables before (water, soil, manure, insects, handling) or after (water, peeling, cutting, packaging, handling) harvest [8]. Furthermore, a growing number of foodborne illness outbreaks have been connected to the consumption of fresh produce and minimally processed fruits and vegetables throughout the food chain [9]. Notably, it has been observed that bacteria that produce extended spectrum β-lactamases (ESBLs), particularly those belonging to the Enterobacteriaceae family (*Klebsiella pneumoniae* and *E. coli*) have been reported in leafy vegetables [10, 11]. Thus, it’s critical to check the microbiological quality on the fresh-cut packed salads.

*K. pneumoniae* is a widespread opportunistic bacterium that causes several human and animal diseases including meningitis, bronchitis, bacteremia, pneumonia, and urinary tract infection [12]. *K. pneumoniae* is considered a common contaminant in many food items including meat, fresh vegetables, and fish so it has been regarded as a significant foodborne pathogen [1].

Resistance to numerous routinely-available drugs has been considered as a public health concern (associated with increased mortality, length of stay and increased cost) due to increased prevalence of extended-spectrum ß-lactamases (ESBLs) and plasmid-mediated AmpCß-lactamase enzyme-producing pathogens [1].ß-lactamases are mainly reported in Gram-negative bacteria, specifically, *Escherichia coli* and *K. pneumoniae* [1]. These bacteria could represent a threat for consumers since they may disseminate during food production and processing [1]. Animals have been identified as the main reservoir for ESBLs-producing microorganisms, and foods may contribute to the spread of resistance to humans through the food chain [1]. Antibiotic-resistant bacteria are disseminated also through the fecal animal waste around farms, slaughterhouses, and meat processing units [4]. Several studies on *K. pneumoniae* isolated from food have reported its multidrug resistant (MDR) phenotype [1, 13]. Clinical management has become more challenging due to the emergence of MDR among *K. pneumoniae* strains that led to increased patient morbidity and mortality [12], therefore it should be taken seriously. So, the WHO, in its global action plan against antimicrobial resistance, has identified that food is one of the potential vehicles for transmission of antimicrobial-resistant bacteria to humans. Further, the human consumption of food carrying resistant bacteria has led to the acquisition of antibiotic-resistant infections [1]. Additionally, the WHO has categorized Enterobacteriaceae that are resistant to carbapenems and third-generation cephalosporins, including *K. pneumoniae*, as critical priority pathogens on its list of antibiotic-resistant bacteria that require new treatments. Also the increased prevalence of ESBLs-producing foodborne bacteria in RTESTF is considered a serious risk [1]. Beside resistance, hypervirulent *K. pneumoniae* (hvKP) has been emerged as a serious clinical pathogen since it causes a plethora of community-acquired infections [14]. Additionally, HvKP utilizes a battery of virulence factors for survival and pathogenesis, such as capsule, siderophores, lipopolysaccharide, fimbriae, outer membrane proteins, and type 6 secretion system [14]. Among the most predominant virulence factors is capsular polysaccharide which increases resistance to phagocyosis [15].Another important virulence determinants in hvKP, the mucoviscosity-associated gene A (*magA*) and the regulator of the mucoid phenotype A (*rmpA*) genes were related to serious invasive infections [15]. Furthermore, siderophores are considered a crucial bacterial virulence factors. The most prevalent siderophore systems are enterobactin (*entB*), aerobactin (*iutA*), yersiniabactin (*ybtS*), and *kfu* gene that encode ferric iron uptake. Likewise, another *K. pneumoniae* virulence determinant is fimbriae which are proteins that can identify a broad range of molecular motifs and guide the bacteria to specific tissue surfaces in the host [12]. Type 1 and type 3 fimbriae (Mrk) grant the attachment of *K. pneumoniae* to cells of the respiratory and urinary tracts [12].

Therefore, in order to prevent these RTESF from spreading to other areas of the environment, food and water screening is necessary.Despite the high risk of transmission to humans through consuming contaminated food, only few studies have been done; hence, the available data is limited. *K. pneumoniae* is recognized as one of the most important Gram-negative opportunistic pathogens, nevertheless, knowledge of the mechanism whereby this bacterium causes different diseases is still unclear and most studies have several limitations because of narrow ranges of virulence factors investigated [16]. Therefore, the current study aimed to assess the prevalence, antimicrobial resistance of *K. pneumoniae* isolated from RTESF in Egypt in addition to scrutinizing the presence of virulence genes.

## Materials and methods

### Samples collection and preparation

The duration of this study was about 2-years (from January 2021 to December 2022) and was conducted in Tanta, Egypt. Sample size of (242) RTEST (green salad) was calculated by epi info 2000 software based on prevalence of outcome (36%) at confidence interval 95% and 90% power of the study. A total of 242 RTEST (green salad) samples were collected from 242 different food suppliers, placed in separate sterile plastic bags, then they were transferred into ice box directly after purchasing and within 24 h, they were moved to the microbiological lab for investigation.Twenty-five (25) gram from each green salad sample were mixed with 225 mL buffered peptone water and then homogenized for 2 min in a laboratory blender Stomacher 400 Circulator (Seward Ltd., Worthing, UK) [1].

### Enumeration of the total bacterial number by viable count technique

The experiment was conducted as previously reported [17]. One mL of each sample homogenate was added to 9 mL sterile distilled water and seven dilutions were made for each sample. Under the aseptic technique, only 0.1 mL of the diluted sample was pipetted out into the sterile nutrient agar plate and distributed gently using L-spreader, and the plate was left to dry and then incubated at 37ºC for 24 h. Finally, the total viable colonies were counted by (LEICA QUEBEC DARKFIELD COLONY COUNTER MODEL 3325) and expressed in CFU/mL.

### Isolationof different organisms and identification of *K. Pneumoniae* Isolates from street food samples

A 100 µl of the ten-fold dilution of each sample homogenate was streaked on the following media: MacConkey agar, Eosin Methylene Blue (EMB)for isolation of enterobacteriaceae (positive and negative lactose fermenters), Mannitol Salt Agar (MSA) for isolation of *Staphylococcus* species and phenol red egg yolk polymyxin agar (PREP) for isolation of *Bacillus *spp (all media were purchased from OXOID, UK).The experiment was carried out as described elsewhere [18, 19]. *K. Pneumoniae *isolates were identified based on Gram’s staining, colony character and different array of standard conventional biochemical tests like Indole, Methyl red, Voges-proskauer, Citrate utilization, Triple sugar iron agar (TSI), Oxidase, Catalase, Capsule, Motility and Urease test [1, 19, 20].

### Antimicrobial susceptibility testing

The antibiotic susceptibility testing was done and interpreted using standard Kirby-Bauer disk diffusion technique according to the Clinical and Laboratory Standards Institute (CLSI) [21]. In this study, a panel of 16 different commercially available antibiotic disks (HiMedia, India) was used. Antibiotics used were cefoperazon (CEP-75 µg), amikacin (AK, 30 µg), amoxicillin/clavulanic acid (AMC, 20/10µg), ampicillin/sulbactam (SAM, 20 µg), cefoxitin (FOX, 30 µg), ceftriaxone (CRO, 30 µg), cefotaxime (CTX, 30 µg), cefuroxime (CXM, 30 µg), co-trimoxazole (COT, 25 µg), chloramphenicol (C, 30 µg), tobramycin (TOB, 15 µg), imipenem (IPM, 10 µg), meropenem (MEM, 10 µg), piperacillin-tazobactam (TPZ, 100/10µg), tetracycline (TE, 30 µg), norfloxacin (NOR, 10 µg). In addition, the minimum inhibitory concentrations (MICs) of imipenem was estimated by using broth microdilution technique according to CLSI [21] and *Escherichia coli* ATCC 25,922 was used as a control.

### Phenotypic detection of extended-spectrum β-lactamases (ESBLs)

The detection of ESBLs was done using double disk synergy test (DDST) as previously reported [22]. The isolates were swabbed onto Mueller Hinton Agar (MHA) and tested for antibiotic resistance to amoxicillin/clavulanic acid (20 µg/10µg), ceftazidime (30 µg/mL), and cefotaxime (30 µg/mL). Upon incubation at 37 °C for 18–24 h ESBLs production was detected by the formation of zone of inhibition around the cephalosporins that increases towards the amoxicillin/clavulanic acid (20 µg/10µg), resulting in synergy formation [22].

### Phenotypic detection of carbapenemases by Triton Hodge Test (THT)

The experiment was performed according to Khalil et al. [23]. Approximately 50 µL of pure Triton X-100 (Sigma-Aldrich, St. Louis, MO, USA) was poured onto the center of MHA plate and immediately coated across the entire plate in 4 to 6 directions. Afterwards, for around 10 min, the plate was left undisturbed until the reagent was entirely absorbed. The test was carried out using meropenem disc (10 µg). Additionally, *K. pneumoniae* ATCC BAA-1705 and ATCC BAA-1706 strains were used as positive and negative controls, respectively.

### Biofilm formation test

The biofilm formation ability of *K. pneumoniae* isolates was tested using the microtiter plate technique as previously described [24]. Briefly,180 mL of Luria-Bertani (LB) broth containing 1% glucose and 20 mL of fresh bacterial culture were added to sterile 96-well flat-based microtiter plates. Sterile LB, supplemented with 1% glucose, was used as a negative control, while *K. pneumoniae* ATCC 13,883 was considered as a positive control. After incubation at 37ºC for 18 h, each well was successively rinsed with phosphate-buffered saline (PBS). Before staining with crystal violet (2%), wells were dried at 60 °C for 1 h. Subsequently, glacial acetic acid 33% (v/v) was used to solubilize the bound dye and the absorbance was estimated at 570 nm (OD570). The experiment was performed three times, and the average reading was considered [24].

Based on the obtained ODs, strains were classified into four groups namely, strong, moderate, weak biofilm producers, and non- biofilm producers [23]. It was reported that the cutoff OD (ODc) was described as the mean OD of the negative control + three standard deviations. The degree of the formed biofilm was reported as follows: strong biofilm formation (OD > 4×ODc), moderate biofilm formation (2×ODc < OD < 4×ODc), weak biofilm formation (ODc < OD < 2×ODc), and non- biofilm formation (OD < ODc) [23].

### Detection of genes encoding β-Lactamases by polymerase chain reaction (PCR)

Two multiplex PCR assays were used for detection of ESBLs genes: one multiplex assay comprised *bla*_TEM_/*bla*_SHV_/*bla*_OXA−__1_ and a second one comprised *bla*_CTX−M_ (including phylogenetic groups 1, 2 and 9) [25]. On the other hand, one uniplex PCR was used for detection of *bla*_CTX_-_M−8/−25_. The genomic DNA was extracted as previously described [26]. Amplification was carried out as follow: initial denaturation at 95 °C for 5 min; 30 cycles of denaturation at 94 °C for 30s, annealing at 56 °C for 30s, and extension at 72 °C for 1 min; and a final extension at 72 °C for 10 min. PCR amplicons were separated electrophoretically on 1.2% agarose gel with ethidium bromide dye and visualized under UV light. For quality control of PCR assay, known control organisms harboring *bla*_CTX_-_M_, *bla*_TEM_, and *bla*_SHV_ were included as positive controls in each run [25]. All primers used are listed in Table 1.

**Table 1:** All primers used to detect the ESBLs genes in this study, their size and concentrations

Target gene	Primer sequence	Target β-lactamase genes	Amplicon size (bp)	Primer conc. (pmol/µl)	Reference
TEM-F TEM-R	5’-CATTTCCGTGTCGCCCTTATTC-3’ 5’-CGTTCATCCATAGTTGCCTGAC-3’	*bla*_TEM_	800	0.4	[25]
SHV-F SHV-R	5’-AGCCGCTTGAGCAAATTAAAC-3’ 5’-ATCCCGCAGATAAATCACCAC-3’	*bla*_SHV_	713	0.4	[25]
OXA-1 F OXA-*1* R	5’-GGCACCAGATTCAACTTTCAAG-3’ 5’-GACCCCAAGTTTCCTGTAAGTG-3’	*bla*_OXA−1_	564	0.4	[25]
CTX-M-1 F CTX-M-1 R	5’-TTAGGAARTGTGCCGCTGYA-3’ 5’-CGATATCGTTGGTGGTRCCAT-3’	*bla*_CTX−MGp1_	688	0.4 0.2	[25] [25]
CTX-M-2 F CTX-M-2 R	5’-CGTTAACGGCACGATGAC-3’ 5’-CGATATCGTTGGTGGTRCCAT-3’	*bla*_CTX MGp2_	404	0.2	[25]
CTX-M-9 F CTX-M-9 R	5’-TCAAGCCTGCCGATCTGGT-3’ 5’-TGATTCTCGCCGCTGAAG-3’	*bla*_CTX MGp9_	561	0.4	[25]
CTX-M-8/25 F CTX-M-8/25 R	5’-AACRCRCAGACGCTCTAC-3’ 5’-TCGAGCCGGAASGTGTYAT-3’	*bla*_CTX Mgroup8/25_	326	0.4	[25]

F: forward primer; R: reverse primer

### Detection of virulence genes of *K. pneumoniae*

Multiplex PCR was used for detection of nine virulence genes in *K. pneumoniae *which were the following, (*ybtS*,* mrkD*,* entB*,* rmpA*,* K2*,*kfu*,* allS*,* iutA*, and *magA)*. Table 2 showed the primer sequence, annealing temperature, the product size, and the concentration of the used primers.The PCR reactions were performed as previously described by [27]. Positive and negative controls were involved in all PCR assays. The amplicons were separated at 100 V for 2 h in a 1.2% (wt/vol) agarose gel containing ethidium bromide [27].

**Table 2:** The primers used to detect the virulence genes in this study, their size and concentrations

Gene	Primer sequence	Function	Annealing Temp (˚C)	Amplicon size (bp)	Primer conc (pmol/µl)	Reference
*ybtS*_F *ybtS*_R	5’-GACGGAAACAGCACGGTAAA-3’. 5’-GAGCATAATAAGGCGAAAGA-3’.	Siderophore	60 °C	242	0.4	[27]
*mrkD*_F *mrkD*_R	5’-AGCTATCGCTGTACTTCCGGCA-3’. 5’-GGCGTTGGCGCTCAGATAGG-3’.	Adhesin type 3 fimbriae	60 °C	340	0.1	[27]
*entB*_F *entB*_R	5’GTCAACTGGGCCTTTGAGCCGTC-3’. 5’-TATGGGCGTAAACGCCGGTGAT–3’.	Siderophore	60 °C	400	0.1	[27]
*rmpA*_F *rmpA*_R	5’-CATAAGAGTATTGGTTGACAG–3’ 5’-CTTGCATGAGCCATCTTTCA–3’	Regulator of mucoid phenotype A	60 °C	461	0.2	[27]
*K2*_F *K2*_R	5’-CAACCATGGTGGTCGATTAG–3’ 5’-TGGTAGCCATATCCCTTTGG–3’	Capsular serotype K2	60 °C	531	0.4	[28]
*kfu*_F *kfu*_R	5’-GGCCTTTGTCCAGAGCTACG–3’ 5’-GGGTCTGGCGCAGAGTATGC–3’	Iron transport and phosphotransferase function	60 °C	632	0.075	[27]
*allS*_F *allS*_R	5’-CATTACGCACCTTTGTCAGC–3’ 5’-GAATGTGTCGGCGATCAGCTT–3’	Allantoin metabolism	60 °C	764	0.1	[27]
*iutA*_F *iutA*_R	5’-GGGAAAGGCTTCTCTGCCAT-3’ 5’-TTATTCGCCACCACGCTCTT-3’	Siderophore	60 °C	920	0.1	[27]
*magA*_F *magA*_R	5’-GGTGCTCTTTACATCATTGC-3’ 5’-GCAATGGCCATTTGCGTTAG-3’	Capsular serotype K1	60 °C	1283	0.3	[29]

### Statistical analysis

**Fig. 1:**
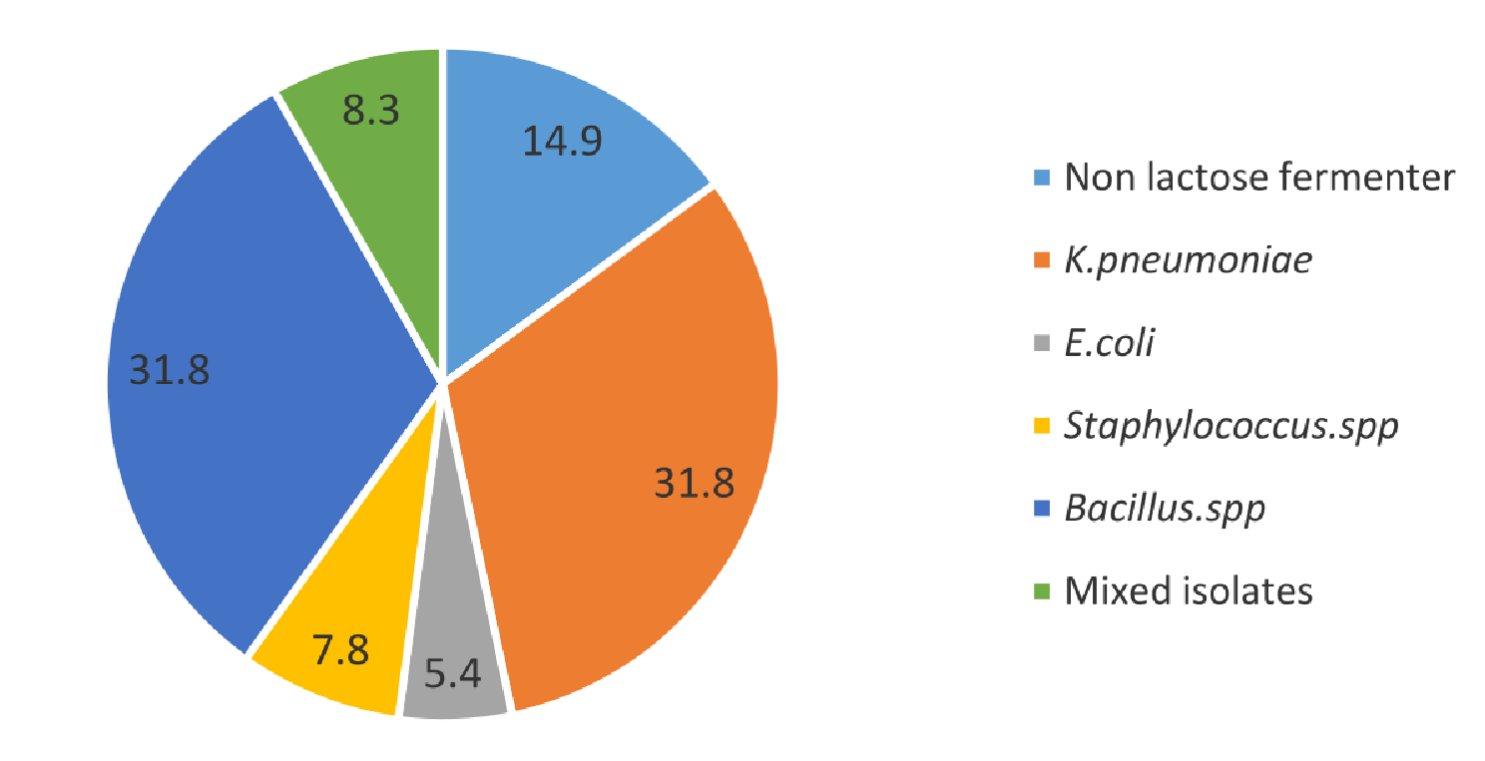
Distribution of different bacterial species among RTESF samples

**Fig. 2:**
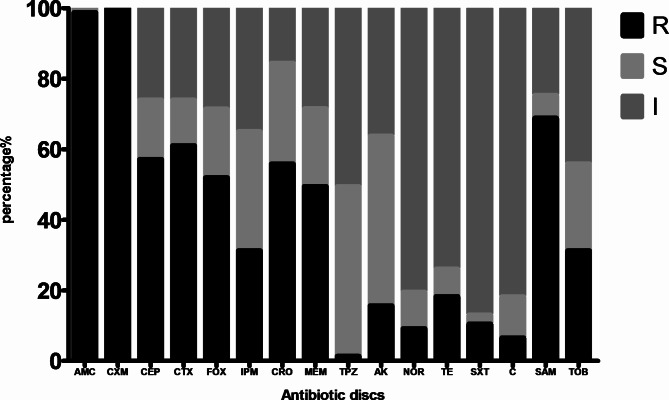
Antibiotic profile of *K. pneumoniae* isolates to tested antibiotics

**Fig. 3:**
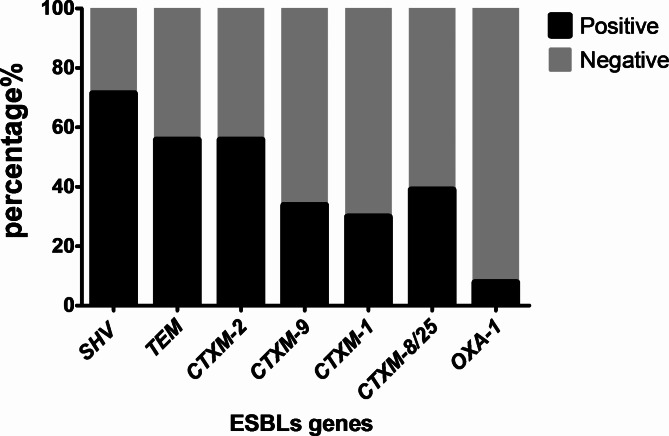
Frequency of different ESBLs genes among *K. pneumoniae* isolates

**Fig. 4:**
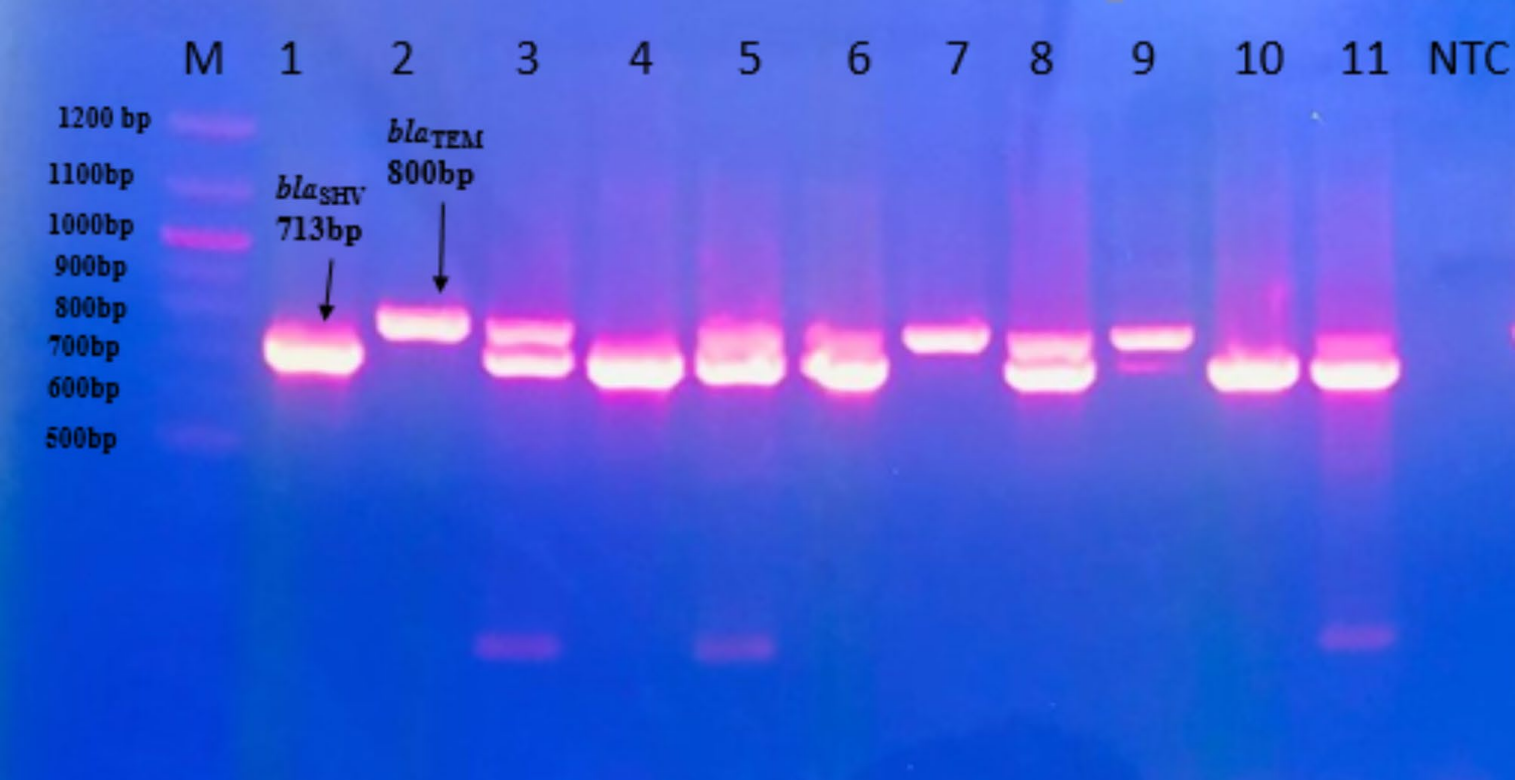
ESBLs genes amplification assays for *K. pneumoniae* by multiplex PCR

Data (antibiotic resistance, biofilm-producers, resistance genes, and virulence genes) analysis was performed using the Statistical Package for the Social Sciences software version 22 (SPSS Inc., Chicago, IL, USA). Data were evaluated using chi square test to compare between more than two qualitative groups. All statistical tests were two-sided. Figures 1, 2, 3 and 4 were prepared using GraphPad Prism software 5.0. The significance of differences was determined at *p* ≤ 0.05.

## Results

### Enumeration of the total bacterial numbers by viable count

Viable count experiment revealed that the bacterial numbers in the collected samples were ranging from 2 × 10^2^ to 3 × 10^9 ^CFU/mL.

### Isolation and identification of different bacterial species from samples

Ninety out of two hundred and forty two (90/242, 37.2%) food samples were Gram-negative lactose fermenting bacteria and (36/242, 14.9%) isolates were Gram-negative non-lactose fermenters. Among ninety Gram-negative positive lactose-fermenters, the most frequent detected pathogen was *K. pneumoniae* (77/90, 85.5%), followed by *E. coli* (13/90, 14.5%). For Gram- positive bacteria, *Staphylococci *spp were present in (19/242, 7.8%) of food samples and other Gram-positive bacterial spp (*Bacillus *spp) were present in (77/242, 31.8%) of the samples. Additionally, it was found that (20/242, 8.3%) of the samples were mixed isolates (Gram-positive bacteria and non-lactose fermenter Gram-negative bacteria).The distribution of bacterial species among street food samples is shown in Fig. 1.

### Antimicrobial susceptibility testing

All *K. pneumoniae* isolates were resistant to cefuroxime, cephradine77/77(100%) and 76/77(98.7%) of the isolates were resistant to amoxicillin/clavulanic acid. The resistance percentages were 53/77(68.8%), 47/77(61%), 44/77(57.1%), 43/77(55.8%), 40/77(51.9%), and 38/77(49.3%) for ampicillin/sulbactam, cefotaxime, ceftriaxone, cefoperazone, cefoxitin, meropenem, respectively. About 24/77(31.2%) of the isolates were resistant to imipenem while 34/77(44.2%) and 28/77(36.4%) of the isolates were susceptible to tobramycin and amikacin, respectively. Co-trimoxazole showed the highest activity as 67/77(87%) of the tested *K.pneumoniae* isolates were co-trimoxazole-susceptible.The sensitivity to chloramphenicol, norfloxacin, tetracycline and piperacillin-tazobactamwere 63/77(81.8%), 62/77(80.5%), 57/77(74%) and 39/77(50.6%), respectively. The results of antibiotic susceptibility tests are shown in Fig. 2. About 21/77(27.3%) of the isolates were resistant to three or more different classes of antibiotics (i.e., MDR).

### Phenotypic detection of ESBLs

Sixty of the isolates (*n* = 60/77, 77.9%) were positive for this test.

### Screening the ability of biofilm formation

Forty one out of 77 isolates (53.2%) showed moderate biofilm formation while 31 of the isolates (40.2%) showed strong biofilm formation. While, only five isolates (6.5%) exhibited weak biofilm formation as shown in Table 3.

**Table 3:** Biofilm formation (strong, moderate, and weak) among *K. pneumoniae* isolates

Biofilm Formation	Number (*n* = 77)	%
Moderate	41	53.2%
Weak	5	6.5%
Strong	31	40.3%

### Phenotypic detection of carbapenemases by THT

Carbapenemases were detected phenotypically using THT. This showed that seven *K. pneumoniae* isolates (9%) of the isolates were carbapenemases producers as indicated by positive THT.

### Molecular detection of ESBLs-genes

It was observed that at least one of these genes was present among *K. pneumoniae *isolates. Interestingly, SHV was the most prevalent gene, since it was identified in 71.4% of the isolates (55/77) while TEM and CTXM-2 were equally identified in 55.8% of the isolates (43/77). Similarly, CTXM-8/25, CTXM-9, and CTXM-1 were found in 38.9% (30/77), 33.76% (26/77), and 29.8% (23/77) of the isolates, respectively. Finally, *bla*_OXA−1_ was identified in 7.79% of the isolates (6/77) as shown in Fig. 3, while Fig. 4 showed multiplex PCR for the detection of some of the studied ESBLs genes.

The first lane represents the DNA marker (100 bp DNA ladder), NTC refer to negative control, and the PCR products were separated on a 1.2% agarose gel. Molecular size marker is shown as lane M. Product size were *bla*_*SHV*_: 713 bp, and bla_*TEM*_: 800 bp.

### Molecular detection of virulence genes

Table 3 illustrated the frequency of the detected nine virulence genes in *K. pneumoniae* isolates.The most prevalent genes were *mrkD *(92.2%) and *K2 *(63.3%) followed by *kfu* and *ybtS *(*51.9%). *Other virulence genes including *entB*,* alls*, and *rmpA* were detected in 49.3%, 36.3%, and 25.9% of the isolates, respectively. The least detected genes were *iutA* and *magA *as they were identified in 22.1% and 9.1% of the isolates, respectively. Additionally, it was found that the presence of *mrkD *(340 bp), *K2 *(531 bp), *allS *(764 bp), and *iutA *(920 bp) genes was significantly associated with strong biofilm producers in comparison to moderate and poor producers with *p*-value < 0.05 Table 4.

**Table 4:** The frequency of the detected nine virulence genes in *K. pneumoniae* isolates

Gene	Size(bp)	No of positive isolates/total isolates (%)	No of negative isolates/total isolates (%)
***mrkD***	340	71/77 (92.2%)	6/77 (7.8%)
***K2***	531	49/77 (63.6%)	28/77 (36.4%)
***Kfu***	242	40/77 (51.9%)	37/77 (48.1%)
***ybtS***	632	40/77 (51.9%)	37/77 (48.1%)
***entB***	400	38/77 (49.4%)	39/77 (50.6%)
***allS***	764	28/77 (36.4%)	49/77 (63.6%
***rmpA***	461	20/77 (26%)	57/77 (74%)
***iutA***	920	17/77 (22.1%)	60/77 (77.9%)
***magA***	1283	7/77 (9.1%)	70/77 (90.9%)

**Table 5:** Biofilm production (moderate, weak, and strong) among *K. pneumoniae* isolates from RTESF in relation to virulence-associated genes. * significant (*p* ≤ 0.05)

Variables (*n* = 77)	Biofilm formation						*p*-value
	Moderate		Weak		Strong		
	No	%	No.	%	No.	%	
*ybtS* 242 bp							
Negative	20	48.8%	4	80%	13	41.9%	0.34
Positive	21	51.2%	1	20%	18	58.1%	
*mrkD* 340							
Negative	6	14.6%	0	0%	0	0%	0.01*
Positive	35	85.4%	5	100%	31	100%	
*entB* 400 bp							
Negative	22	53.7%	4	80%	13	41.9%	0.21
Positive	19	46.3%	1	20%	18	58.1%	
*rmpA* 461 bp							
Negative	33	80.5%	5	100%	19	61.3%	0.06
Positive	8	19.5%	0	0%	12	38.7%	
*K2* 531 bp							
Negative	19	46.3%	1	20%	8	25.8%	0.04*
Positive	22	53.7%	4	80%	23	74.2%	
*Kfu* 632 bp							
Negative	23	56.1%	3	60%	11	35.5%	0.06
Positive	18	43.9%	2	40%	20	64.5%	
*allS* 764 bp							
Negative	29	70.7%	5	100%	15	48.4%	0.04*
Positive	12	29.3%	0	0%	16	51.6%	
*iutA* 920 bp							
Negative	37	90.2%	4	80%	19	61.3%	0.003*
Positive	4	9.8%	1	20%	12	38.7%	
*magA* 1283 bp							
Negative	38	92.7%	5	100%	27	87.1%	0.30
Positive	3	7.3%	0	0%	4	12.9%	

In the current study, thirty different virulence profiles were detected among *K. pneumoniae* isolates as shown in table 5. It was found that among the most prevalent virulence profiles were the *mrkD* + *K2* genes (59.7%) in *K. pneumoniae* isolates followed by *mrkD* + *entB* genes (46.7%) simultaneously. Moreover, data obtained revealed two profiles for *K. pneumoniae* isolates harbored seven virulence genes simultaneously as follows: *ybtS* + *mrkD* + *entB* + *rmpA* + *K2* + *Kfu* + *alls* (3.9%) and *mrkD* + *entB* + *rmpA* + *K2* + *Kfu* + *allS* + *iutA *(2.5%). The full detailed profiles demonstrating the coexistence of virulence associated genes among *K. pneumoniae* isolates are listed in Table 6.

**Table 6:** Coexistence of virulence associated genes among *K. pneumoniae* isolates

Virulence profile	Virulence genes detected	No of isolates (%)
1	*mrkD*, *entB*	36/77(46.7)
2	*rmpA*, *magA*	2/77(2.5)
3	*entB*, *iutA*, *Kfu*	9/77(11.6)
4	*ybtS*, *mrkD*	35/77(45.4)
5	*ybtS*, *mrkD*, *entB*,* K2*	12/77(15.5)
6	*ybtS*, *mrkD*, *entB*, *rmpA*	5/77(6.5)
7	*entB*, *Kfu*, *allS*, *iutA*	6/77(7.8)
8	*ybtS*, *mrkD*, *entB*, *Kfu*	10/77(13)
9	*ybtS*, *mrkD*, *rmpA*, *K2*, *Kfu*	5/77(6.5)
10	*K2* + *Kfu* + *mrkD* + *magA*	2/77(2.5)
11	*ybtS*, *mrkD*, *allS*, *iutA*	9/77(11.6)
12	*ybtS*, *mrkD*, *rmpA*, *Kfu*	8/77(10.4)
13	*ybtS*, *entB*, *iutA*, *Kfu*	6/77(7.8)
14	*ybtS*, *mrkD*, *entB*, *rmpA*, *K2*, *Kfu*	4/77(5.2)
15	*ybtS*, *mrkD*, *K2*	25/77(32.5)
16	*ybtS*, *mrkD*, *K2*, *Kfu*	17/77(22)
17	*ybtS*, *mrkD*, *rmpA*, *K2*	5/77(6.5)
18	*ybtS*, *mrkD*, *entB*, *rmpA*, *K2*, *Kfu*, *allS*	3/77(3.9)
19	*mrkD*, *entB*, *rmpA*, *K2*, *Kfu*, *allS*	6/77(7.8)
20	*mrkD*, *entB*, *rmpA*, *K2*, *Kfu*, *allS*, *iutA*	2/77(2.5)
21	*Kfu*, *allS*, *iutA*	10/77(13)
22	*mrkD*, *rmpA*, *iutA*	7/77(9.1)
23	*mrkD*, *rmpA*, *K2*	11/77(14.3)
24	*mrkD*, *rmpA*, *K2*, *Kfu*, *allS*	7/77(9.1)
25	*entB*, *K2*	27/77(35)
26	*rmpA*, *K2*	12/77(15.6)
27	*mrkD*, *K2*	46/77(59.7)
28	*ybtS*, *K2*	28/77(36.4)
29	*K2*, *Kfu*	31/77(40)

## Discussion

There are infrequent studies on microbiological investigations on ready to eat (RTE) salads with dressings/sauces, these studies were applied only to raw vegetables or salad blends [30]. Additionally, the microbiological investigations were also applied to RTE products in general, since it was reported that there are many sources of microbial contaminations in RTE foods, among these sources the ingredients as well as the processing and handling could lead to cross contamination [31]. The current study showed a significant increase in the microbial contamination among 242 RTE fresh salads where the total viable bacterial count in the collected samples were ranging from 2 × 10^2^ to 3 × 10^9^ CFU/mL.Our results are in agreement with the study from Bangladesh [32] and Poland [30].

There are several ways that RTESF can become contaminated. In the current this study, it was observed that these items are not heated and are only partially covered before being served. Additionally, street food vendors use their hands to serve food. Coins and tickets that are unclean and contaminated are taken or given by these same hands, the same observation was reported in Ghana [33]. Additionally, the water often used by street foods vendors is extremely unclean and salads are frequently contaminated with pathogenic microorganisms due to mishandling of raw vegetables, either during salads preparation or from environment as soils typically harbor abundant microorganisms [34]. These different practices may be induced the contamination of street foods. The lack of hygiene in the commercialization process of street foods leads to the microbial foodborne disease that can reach one or more people at a time.

Recently it has been reported that *K. pneumoniae* is the main cause of foodborne outbreaks in different countries [35]. The present study reported a high prevalence of *K. pneumoniae *(31.8%) in RTE fresh salads sampleswhich was higher than previously reported in Egypt [36]. Our finding indicates that the prevalence of *K. pneumoniae* in RTESF is substantially increasing and food contamination with *K. pneumoniae* is common in Egypt. Data obtained were in line with many previous studies including those from India (27.12%) [1], Dominican Republic, India, Thailand, and Vietnam (43.3%) [37], Malaysia (32%) [13], Nigeria (20.24%) [38] and Kenya (29%) [39]. On contrary, our finding were different from those reported from China (9.9%) [40] and Spain (5.6%) [41].With regard to other pathogens, *E. coli* was detected in low abundance (5.4%)in this study as compared to an Indian study (22.88%) [1] also prevalence of *Staphylococci *species was in very low abundance when compared to a study from Thailand [42]. Furthermore, the prevalence of Gram negative non-lactose fermenters was lower than that reported by a study from Ethiopia [43]. The current study showed that almost all *K. pneumoniae* isolates were resistant to cefuroxime and amoxicillin/clavulanic acid and showed higher resistance to third-generation cephalosporins including cefotaxime (61%), cefoperazone (57.1%), and ceftriaxone (55.8%). Our data revealed that the most effective antibiotic was cotrimoxazole (87%) followed by chloramphenicol (81.8%) and norfloxacin (80.5%) which is in concordance with a study by Zhang et al. [35]. Here, we reported that resistance ratios to imipenem, and meropenem were 31.2% and 49.4%, respectively which could be attributed, at least partly, to production of carbapenemases which is in line with Abdel-Rhman et al. [36].The resistance toward imipenem (31.2%) and meropenem (49.4%) should be considered seriously as these antibiotics are classified as lifesaving drugs which are used in treating serious infections [1]. The high rates of antimicrobial resistance detected in this study could be attributed to the lack of strict policies that govern the use of antibiotics in Egypt.Various mechanisms are likely to be involved in such resistance including AmpC or ESBLs production with porin loss, carbapenemase production, and/or metallo-β-lactamase production [44].

This study reported that 77.9% of *K. pneumoniae* were ESBLs-producers and this is in line with previous studies from Italy, Iran and South Korea with prevalence rates of 83.3%, 71.4% [45] and 84.2% [46], respectively. It was reported that the percentages of ESBLs-producing *K. Pneumoniae *vary among countries with high percentages reported in Arabian countries [47, 48]. Moreover, studies from Egypt and other countries are scarce regarding the prevalence of ESBLs-producing *K. pneumoniae* from RTESF. Currently, most of the data focus on *E. coli* and other major foodborne pathogens from various sources, thereby lead to underestimating *K. pneumoniae* as a potential organism prevalent in RTESF, therefore, our finding highlights the importance to investigate ESBLs-producing *K. pneumoniae* in RTESF. All phenotypic methods, used in this study, to detect ESBLs and carbapenemases production were unable to differentiate between types or families of each class where all β-lactamase classes now present immediate clinical impact [49]. All ESBLs genes (*bla*_CTX−M_, *bla*_SHV_, and *bla*_TEM_) tested in the current work were class A which are considered the most clinically significant ESBLs variants ß-lactamases are the primary cause of β-lactams resistance among Enterobacteriaceae. Here, the most predominant was *bla*_SHV _(71.4%) which is in line with previous reportsfrom Egypt [36] and Iran [45]. On contrary, our results are slightly different with the study of Iseppi et al. where the resistant genes mainly belonged to the CTX-M family [18]. Additionally, our results are also different to the study of Maina et al. where TEM was the most prevalent (55%) gene [39]. In the current study it was found that TEM and CTXM-2 were equally present in isolates which is similar to the study fromIran [45]. Furthermore, CTXM-8/25, CTXM-9, and CTXM-1 were detected in 38.9%, 33.76%, and 29.8% of isolates, respectively while OXA-1 was identified in 7.79% of the isolates. The seresults are contrary to the study of Maina et al. where OXA-1 was present in 39% of the isolates [39]. Furthermore, an Indian study reported high prevalence of TEM (40.68%), followed by CTX (32.20%) and SHV (10.17%) [1].

Biofilm formation by *K. pneumoniae *is crucial in facilitating evasion of host defense mechanisms, communication between bacterial cells and protection against antibiotic action [50].In this study, 93.5% of the isolates were detected phenotypically as biofilm producers (53.2% of them were moderate and 40.3% were strong) and this is in line with a previous study from Egypt [51] and *mrkD *was genotypically detected in 92.2% of the isolates. Here, we thought to investigate nine virulence genes of *K. pneumoniae* namely, *ybtS*,* mrkD*,* entB*,* rmpA*,* K2*,* kfu*,* alls*,* iutA* and *magA*by PCR. This showed that *K1 *was detected in 9.1% of isolates similar to an Iranian study (11.2%) [52] and lower than an Egyptian study (28.5%) [53]. Likewise, *K2 *was detected in 63.6% of isolates which is higher than previous studies [53, 54]. Afterwards, siderophores were investigated and this showed that *entB* was detected in 49.4% of the isolates which is in agreement with an Egyptian study (68%) [51]. However *entB* is only associated with virulence when it occurs in association with *iutA* or *kfu* [55]. In the current study, 9(11.6%) of *K. pneumoniae* carried these three genes together. For *entB* in combination to *iutA* gene was found only in 11(14.3%) of the isolates while the co-existence of *entB* and *kfu* together was 24(31.2%) among the isolates. These result is lower than that of Naga et al. where the existence of the genes encoding *entB* in combination with *iutA*and *kfu* was found in 66% and 68%, respectively [51]. Likewise, *iutA* was detected in 22.1% of the isolates which is in line with a previous study from Egypt (34%) [51]. Additionally, there was a significant correlation between biofilm-production and *iutA* gene (*p*-value = 0.003).Regarding *ybtS*, encodingYersiniabactin, it was detected in 51.9% of isolates compared to a Chinese study (95.9%) [56] and Iranian study (39%) [57]. For iron acquisition system, *kfu *was detected in 51.9%of isolates which is lower than an Egyptian study (100%) [51]. The most prevalent virulence genes was *mrkD *(92.2%) which is in line with previous studies [51, 56].As per *rmpA*, a putative virulence factor that has been found to be associated with highly virulent *K. pneumoniae* [15], a lower prevalence was (26%) detected compare with others (52%) [51]. Here, coexistence of *K1* and *rmpA* were only detected in 2.5% of the isolates, while 15.6% of the isolates coharbored *rmpA* and *K2*. Data obtained revealed that *allS*, an activator of the allantoin regulon [35], was detected in 36.4% of isolates which is higher than reported elsewhere [35, 56]. It was observed that there was a significant correlation between *mrkD* and resistance to cefoxitinwith *p*-value < 0.05. Likewise *entB* showed a significant correlation with resistance to imipenem, and ceftriaxone. For *K2* gene there was a significant correlation with resistance to cefoperazone, while Kfu showed a significant correlation with resistance to imipenem, and meropenem. Finally, for *allS* it showed significant correlation with resistance to cefoxitin, and imipenem and for *iutA* there was significant correlation with resistance to meropenem with *p*-value < 0.05 as shown in supplementary table-1. Therefore, the presence of virulence factors and antibiotic resistance were, at least partly, directly correlated and this observation is in accordance to a previously published work from Egypt [26].

## Conclusion

In conclusion, to our knowledge the present study is considered the first report in Egypt that investigated RTE fresh green salad. We reported a highly resistant profile as well as a hyper virulent strain profile of *K. pneumoniae *isolates which were recovered from RTESF which represents a good reservoir of resistant *K. pneumoniae* isolates. This represents a major public health concern, therefore a restricted control of the emergence and the transmission of these isolates is needed. This can be attained by developing more prevention strategies on processing and handling this type of food.

## Electronic supplementary material

Below is the link to the electronic supplementary material.

Supplementary Material 1

## Data Availability

All the datasets generated or analyzed during this study are included in this manuscript.

## Abbreviations

RTESF: Ready-To-Eat-Street-Foods
*K. pneumoniae*: *Klebsiella pneumoniae*
ESBLs: Extended-spectrum ß-lactamases
MDR: Multidrug resistant
WHO: World Health Organization
FBDs: Foodborne diseases
hvKP: Hypervirulent K. pneumoniae
magA: Mucoviscosity-associated gene A
entB: Enterobactin
rmpA: Regulator of the mucoid phenotype A
iutA: Aerobactin
ybtS: Yersiniabactin
EMA: Eosin Methylene Blue
MSA: Mannitol Salt Agar
PREP: Phenol red egg yolk polymyxin agar
CLSI: Clinical and Laboratory Standards Institute
CEP-75µg: Cefoperazon
AK, 30µg: Amikacin
TOB, 15µg: Tobramycin
SAM, 20µg: Ampicillin/sulbactam
AMC, 20/10µg: Amoxicillin/clavulanic acid
FOX, 30µg: Cefoxitin
CXM, 30µg: Cefuroxime
CTX, 30µg: Cefotaxime
CRO, 30µg: Ceftriaxone
COT, 25µg: Co-trimoxazole
C, 30µg: Chloramphenicol
TE, 30µg: Tetracycline
PCR: Polymerase chain reactions
IPM, 10µg: Imipenem
MEM, 10µg: Meropenem
TPZ, 100/10µg: Piperacillin-tazobactam
NOR, 10µg: Norfloxacin
MICs: Minimum inhibitory concentrations
DDST: Double disc synergy test
THT: Triton hodge Test
MHA: Mueller hinton agar
OD: Optical denisty
SPSS: Statistical Package for the Social Sciences software version
