# Supplementary material for: Phenotypic and genotypic screening of multidrug resistant Klebsiella pneumoniae isolated from ready to eat street food in Tanta, Egypt
Source: BMC Microbiol. 2025 Feb 5;25:65. doi: 10.1186/s12866-025-03769-z (PMC11796132; doi:10.1186/s12866-025-03769-z)
Supplement: Supplementary file 1 — Supplementary Material 1 [file 12866_2025_3769_MOESM1_ESM.docx]

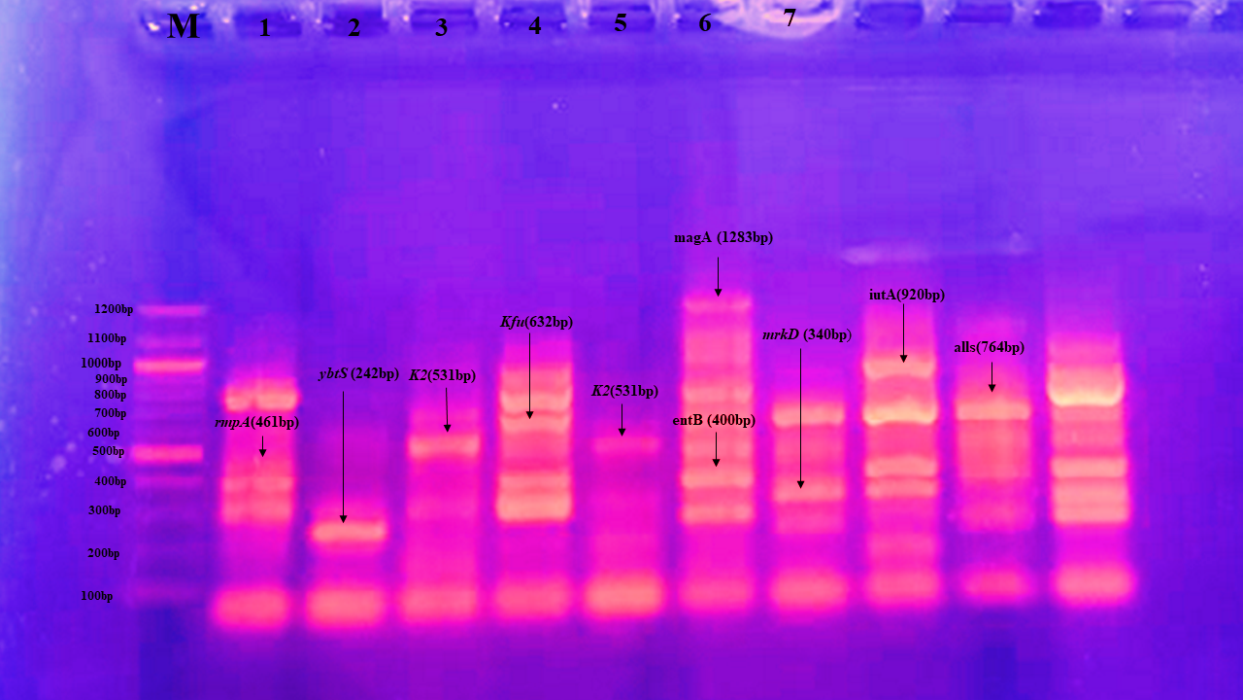


**Supplementary Fig.1**: Virulence gene amplification assays for *K. pneumoniae* by multiplex PCR.

The first lane represents the DNA marker (100bp DNA ladder) and the PCR products were separated on a 1.2% agarose gel. Molecular size marker is shown as lane M. Product size were *yptS*: 242bp, *mrkD*: 340bp, *entB*: 400bp, *rmpA*: 461bp, *k2*: 531bp, *kfu*: 632bp: *allS*: 764bp, *iutA*: 920bp, *magA*: 1283bp.

**Supplementary table 1A**: **Statistical analysis regarding the relationship between biofilm and virulence genes**

| Variables  (n=77) | Biofilm formation | | | | | | p-value |
| --- | --- | --- | --- | --- | --- | --- | --- |
|  | **Moderate** | | **Weak** | | **Strong** | |  |
|  | **No** | **%** | **No.** | **%** | **No.** | **%** |  |
| *ybts* 242 bp | | |  |  |  |  |  |
| Negative | 20 | 48.8% | 4 | 80% | 13 | 41.9% | 0.34 |
| Positive | 21 | 51.2% | 1 | 20% | 18 | 58.1% |  |
| mrkD 340 | | |  |  |  |  |  |
| Negative | 6 | 14.6% | 0 | 0% | 0 | 0% | **0.01*** |
| Positive | 35 | 85.4% | 5 | 100% | 31 | 100% |  |
| entB 400 bp | | |  |  |  |  |  |
| Negative | 22 | 53.7% | 4 | 80% | 13 | 41.9% | 0.21 |
| Positive | 19 | 46.3% | 1 | 20% | 18 | 58.1% |  |
| rmpA 461 bp | | |  |  |  |  |  |
| Negative | 33 | 80.5% | 5 | 100% | 19 | 61.3% | 0.06 |
| Positive | 8 | 19.5% | 0 | 0% | 12 | 38.7% |  |
| K2 531 bp |  |  |  |  |  |  |  |
| Negative | 19 | 46.3% | 1 | 20% | 8 | 25.8% | **0.04*** |
| Positive | 22 | 53.7% | 4 | 80% | 23 | 74.2% |  |
| Kfu 632 bp |  |  |  |  |  |  |  |
| Negative | 23 | 56.1% | 3 | 60% | 11 | 35.5% | 0.06 |
| Positive | 18 | 43.9% | 2 | 40% | 20 | 64.5% |  |
| allS 764 bp |  |  |  |  |  |  |  |
| Negative | 29 | 70.7% | 5 | 100% | 15 | 48.4% | **0.04*** |
| Positive | 12 | 29.3% | 0 | 0% | 16 | 51.6% |  |
| iutA 920 bp |  |  |  |  |  |  |  |
| Negative | 37 | 90.2% | 4 | 80% | 19 | 61.3% | 0.**003*** |
| Positive | 4 | 9.8% | 1 | 20% | 12 | 38.7% |  |
| magA 1283 bp |  |  |  |  |  |  |  |
| Negative | 38 | 92.7% | 5 | 100% | 27 | 87.1% | 0.30 |
| Positive | 3 | 7.3% | 0 | 0% | 4 | 12.9% |  |

**Supplementary table 1B**: **Statistical analysis regarding the relationship between biofilm and antibiotic resistancegenes**

| Variables  (n=77) | Biofilm formation | | | | | | p-value |
| --- | --- | --- | --- | --- | --- | --- | --- |
|  | **Moderate** | | **Weak** | | **Strong** | |  |
|  | **No** | **%** | **No.** | **%** | **No.** | **%** |  |
| OXA-1 564bp | | |  |  |  |  |  |
| Negative | 38 | 92.7% | 5 | 100% | 28 | 90.3% | 0.47 |
| Positive | 3 | 7.3% | 0 | 0% | 3 | 9.7% |  |
| SHV 713bp | | |  |  |  |  |  |
| Negative | 10 | 24.4% | 2 | 40% | 10 | 32.3% | 0.27 |
| Positive | 31 | 75.6% | 3 | 60% | 21 | 67.7% |  |
| TEM 800bp | | |  |  |  |  |  |
| Negative | 20 | 48.8% | 1 | 20% | 13 | 41.9% | 0.31 |
| Positive | 21 | 51.2% | 4 | 80% | 18 | 58.1% |  |
| CTXM-2(404) | | |  |  |  |  |  |
| Negative | 20 | 48.8% | 3 | 60% | 11 | 35.5% | 0.16 |
| Positive | 21 | 51.2% | 2 | 40% | 20 | 64.5% |  |
| CTXM-9(561) |  |  |  |  |  |  |  |
| Negative | 24 | 58.5% | 5 | 100% | 22 | 71% | 0.15 |
| Positive | 17 | 41.5% | 0 | 0% | 9 | 29% |  |
| CTXM-1(688) |  |  |  |  |  |  |  |
| Negative | 31 | 75.6% | 4 | 80% | 19 | 61.3% | 0.12 |
| Positive | 10 | 24.4% | 1 | 20% | 12 | 38.7% |  |
| CTXM-8/25(326) |  |  |  |  |  |  |  |
| Negative | 27 | 65.9% | 3 | 60% | 17 | 54.8% | 0.21 |
| Positive | 14 | 34.1% | 2 | 40% | 14 | 45.2% |  |

**Supplementary table 2**: **The correlation between the virulence genes and different antibiotics in *K. pneumoniae* isolates**

| Antibiotic (n=77) | Positive virulence genes | | | | | | | | |
| --- | --- | --- | --- | --- | --- | --- | --- | --- | --- |
|  | ***ybts* 242 bp** | ***mrkD* 340** | ***entB* 400 bp** | ***rmp*A 461 bp** | ***K2* 531 bp** | ***Kfu* 632 bp** | ***allS* 764 bp** | ***iutA* 920 bp** | ***magA* 1283 bp** |
|  | **No. (%)** | **No. (%)** | **No. (%)** | **No. (%)** | **No. (%)** | **No. (%)** | **No. (%)** | **No. (%)** | **No. (%)** |
| AMC | 40(100%) | 70(98.6%) | 38(100%) | 20(100%) | 49(100%) | 40(100%) | 28(100%) | 17(100%) | 7(100%) |
| CXM | 40(100%) | 71(100%) | 38(100%) | 20(100%) | 49(100%) | 40(100%) | 28(100%) | 17(100%) | 7(100%) |
| CEP | 25(62.5%) | 40(56.3%) | 21(55.3%) | 12(60%) | **30(61.2%)*** | 25(62.5%) | 18(64.3%) | 13(76.5%) | 4(57.1%) |
| CTX | 25(62.5%) | 42(59.2%) | 25(65.8%) | 13(65%) | 32(65.3%) | 27(67.5%) | 19(67.9%) | 11(64.7%) | 5(71.4%) |
| FOX | 24(60%) | **34(47.9%)*** | 24(63.2%) | 11(55%) | 28(57.1%) | 24(60%) | **15(53.6%)*** | 11(64.7%) | 6(85.7%) |
| IPM | 13(32.5%) | 21(29.6%) | **13(34.2%)*** | 7(35%) | 16(32.7%) | **16(40%)**** | **10(35.7%)*** | 3(0.15%) | 3(42.9%) |
| CRO | 25(62.5%) | 38(53.5%) | **24(63.2%)*** | 11(55%) | 29(59.2%) | 24(60%) | 17(60.7%) | 7(41.2%) | 5(71.4%) |
| MEM | 23(57.5%) | 33(46.5%) | 19(50%) | 9(45%) | 26(53.1%) | **23(57.5%)*** | 13(46.4%) | **4(23.5%)*** | 3(42.9%) |
| TPZ | 1(2.5%) | 1(1.4%) | 0(0%) | 0(0%) | 1(2%) | 1(2.5%) | 0(0%) | 0(0%) | 0(0%) |
| AK | 6(15%) | 10(14.1%) | 9(23.7%) | 4(20%) | 8(16.3%) | 9(22.5%) | 5(17.9%) | 1(5.9%) | 1(14.3%) |
| NOR | 3(7.5%) | 7(9.9%) | 5(13.2%) | 1(5%) | 6(12.2%) | 3(7.5%) | 5(17.9%) | 2(11.8%) | **3(42.9%) **** |
| TE | 9(22.5%) | 14(19.7%) | 8(21.1%) | 4(20%) | 9(18.4%) | 8(20%) | 7(25%) | 4(23.5%) | 2(28.6%) |
| SXT | 4(10%) | 8(11.3%) | **7(18.4%)*** | 2(10%) | 5(10.2%) | 6(15%) | **5(17.9%)*** | 2(11.8%) | 1(14.3%) |
| C | 3(7.5%) | 5(7%) | 2(5.3%) | 1(5%) | 3(6.1%) | 2(5%) | 2(7.1%) | 0(0%) | 1(14.3%) |
| SAM | 31(77.5%) | 48(67.6%) | 28(73.7%) | 14(70%) | 33(67.3%) | 26(65%) | 18(64.3%) | 11(64.7%) | **4(57.1%)*** |
| TOB | 16(40%) | **19(26.8%)*** | 14(36.8%) | 3(15%) | 14(28.6%) | 12(30%) | 5(17.9%) | 2(11.8%) | 4(57.1%) |

***statistical significance difference p-value <0.05**

**** High statistical significance difference p-value <0.01**

**Supplementary table 3: Antibiotypes of K. Pneumoniae isolates based on antibiogram pattern**

| **Antibiotype** | **Antibiogram pattern** | **No. of isolates (%)** |
| --- | --- | --- |
| A1 | AMC | 76(98.7) |
| A2 | AMC, CXM | 76(98.7) |
| A3 | AMC, CXM, CEP | 43(55.8) |
| A4 | AMC, CXM, CEP, CTX | 30(38.9) |
| A5 | AMC, CXM, CEP, CTX, FOX | 17(22.1) |
| A6 | AMC, CXM, CEP, CTX, FOX, C | 1(1.3) |
| A7 | AMC, CXM, CEP, CTX, FOX, SAM | 13(16.9) |
| A8 | AMC, CXM, CEP, CTX, FOX, IPM | 6(7.8) |
| A9 | AMC, CXM, CEP, CTX, FOX, IPM, CRO | 4(5.2) |
| A10 | AMC, CXM, CEP, CTX, FOX, SAM, TOB | 7(9.1) |
| A11 | AMC, CXM, CEP, CTX, FOX, IPM, CRO, MEM | 4(5.2) |
| A12 | AMC, CXM, CEP, CTX, FOX, IPM, CRO, MEM, AK | 2(2.6) |
| A13 | AMC, CXM, CEP, CTX, FOX, IPM, CRO, MEM, AK, SAM | 2(2.6) |
| A14 | AMC, CXM, CEP, CTX, FOX, IPM, CRO, MEM, AK, SAM, TOB | 2(2.6) |
| A15 | AMC, CXM, CTX, IPM, CRO, MEM, AK, NOR, TE, SXT, SAM, TOB | 1(1.3) |
